# Value-based payment for high-cost treatments in Singapore: a qualitative study of stakeholders’ perspectives

**DOI:** 10.1017/S0266462324000217

**Published:** 2024-04-17

**Authors:** Diana Beatriz Bayani, Hwee Lin Wee

**Affiliations:** Saw Swee Hock School of Public Health, National University of Singapore, Singapore, Singapore

**Keywords:** value-based payment, risk-sharing agreement, qualitative inquiry, health technology assessment, health financing

## Abstract

**Objectives:**

The rising costs of drugs have necessitated the exploration of innovative payment methods in healthcare systems. Risk-sharing agreements (RSAs) have been implemented in many countries as a value-based payment mechanism to manage the uncertainty associated with expensive technologies. This study aimed to investigate stakeholder perspectives on value-based payment in the Singaporean context, providing insights for future directions in health technology assessment and financing.

**Methods:**

This descriptive qualitative inquiry involved participant interviews conducted between October 2021 and April 2022. Thematic analysis was conducted in two phases to analyze the interview transcripts.

**Results:**

Seventeen respondents participated in the study, and five key themes emerged from the analysis. Stakeholders viewed RSAs as moderately positive, despite limited experience with them. They emphasized the importance of clearly defining objectives and establishing transparent criteria for implementing these schemes. The current data infrastructure was identified as both a barrier and facilitator, as RSAs impose administrative burdens. To successfully implement these payment mechanisms, capacity building, and effective stakeholder engagement that fosters mutual trust and cocreation are crucial.

**Conclusion:**

This study confirms previously identified barriers and facilitators to successful RSA implementation while contextualizing them within the Singaporean setting. The findings suggest that value-based payment has the potential to address uncertainty and improve access to healthcare technologies, but these barriers must be addressed for the schemes to be effective.

## Introduction

Given the increasing cost of healthcare, paying for highly expensive therapies has become a significant challenge to healthcare payers. The high cost of drugs is common in the fields of oncology, hematology, and rare diseases, where the monthly cost of treatment can easily reach thousands of dollars (1;2). However, the high cost of drugs does not always correspond to their value, especially when there is considerable uncertainty surrounding their long-term benefits, particularly with drugs approved through accelerated tracks by regulatory agencies (3;4).

To tackle the issue of costly therapies, healthcare systems must prioritize their allocation of resources. Health technology assessment (HTA) processes have proven helpful in assessing the value of drugs through cost-effectiveness analyses (CEAs), which can guide pricing and payment mechanisms for these treatments (5;6). In situations where uncertainty is high, managed entry schemes or risk-sharing agreements (RSAs) have been implemented to distribute the risk between payers and drug companies. These agreements may take the form of outcome-based or financial-based schemes, depending on their design (7). In Singapore, the Agency for Care Effectiveness (ACE) has led HTA efforts since 2015, with a value-based pricing strategy conducted in parallel (8).

Recently, ACE has implemented a new process allowing drug companies to prepare and submit evidence to request a subsidy listing. This is a departure from the existing procedure, in which ACE’s technical team conducts evaluations in-house with limited involvement from the manufacturers (9). Under this new process, RSAs may be proposed. Initially focused on oncology drug applications for inclusion in the Ministry of Health’s Cancer Drug List between 2021 and 2023, the ACE initiative has broadened its scope to include selected non-cancer drugs starting from 2024 onwards.

This study aims to explore the perspectives of relevant stakeholders within Singapore’s healthcare system regarding novel payment mechanisms for high-cost technologies, with the objective of maximizing value. Specifically, it seeks to achieve the following objectives: (i) identify perceptions, barriers, and facilitators associated with implementing RSAs, (ii) describe feasible and ideal schemes for implementation and determine priority areas of application, and (iii) gather insights into plans for capacity building. While ACE currently only consider price–volume or financial-based RSAs, it is crucial to identify the barriers and facilitators to the successful design and implementation of outcome-based schemes to inform the future direction of this critical aspect of HTA and financing. Through a qualitative analysis of stakeholder perspectives, this study aims to contribute to a deeper understanding of the potential benefits and challenges of outcome-based payment approaches within Singapore’s healthcare system.

## Methods

### Study Design

This study employed a qualitative descriptive methodology based on grounded theory (10–12). The qualitative descriptive methodology aimed to provide a detailed and straightforward account of the phenomenon or experience under investigation (13;14). Simultaneously, grounded theory sought to develop a theory based on systematically collected and analyzed data (15). By using both approaches, this study aimed to gain a comprehensive understanding of stakeholders’ sentiments regarding RSAs, specifically, the outcome-based types.

### Recruitment of Participants

Purposive sampling was employed to recruit stakeholders who were likely to be involved in RSAs (16). Invitations were sent via email to preidentified stakeholder groups, with a focus on industry and government representatives. Respondents were also asked to refer other appropriate participants through snowball sampling until data saturation was achieved. Saturation occurs when there is redundancy in the data collected and the interviews cease to generate new codes or themes (17;18).

### Data Collection

A discussion guide was developed based on findings from a systematic review(19) and was reviewed by two additional researchers (see Supplementary Material). Semistructured, one-on-one, in-depth interviews were conducted via teleconference, recorded, and transcribed by the lead researcher, who possesses experience and training in qualitative data collection and analysis. A modified version of the discussion guide was provided to one respondent who declined an interview and preferred to provide a documented response. The interviewer took field notes, which were analyzed reflexively and incorporated into the results.

The interview guide explored the following topics: (i) participants’ knowledge and understanding of value-based or outcome-based payments, (ii) interest in exploring schemes in the Singaporean context, (iii) barriers to implementation, (iv) readiness and feasibility of implementing schemes, (v) applicability of different schemes across various technologies, and (vi) capacity needs and requirements for preparation and sustainability. To ensure a standardized definition in the discussion of the first topic, the interviewer provided a definition of RSAs based on the taxonomy described in the International Society for Pharmacoeconomics and Outcomes Research (ISPOR) Task Force report on performance-based RSAs (7).

### Data Analysis

Before coding the data, the interview transcripts were read, reread, and organized based on the key topics in the discussion guide. These transcripts were then coded using NVivo(20), and a codebook was developed and refined through validation by another researcher. Thematic analysis occurred in two phases: (i) themes were reviewed alongside coded excerpts, and (ii) themes were analyzed in conjunction with the entire dataset. Deductive coding was applied based on the initial structure of the discussion guide, supplemented by inductive coding when additional insightful feedback emerged from the data. Final themes were organized according to the study objectives, which were to (i) identify perceptions, barriers, and facilitators to implementing RSAs in Singapore, (ii) describe feasible ideal schemes and priority areas for application, and (iii) develop plans for capacity building.

### Ethics Approval

Formal informed consent was obtained prior to conducting the interviews, and participants were informed of their right to withdraw from the study at any time without penalty. To protect participants’ confidentiality, all data were anonymized. The study received ethical approval from the Saw Swee Hock School of Public Health Departmental Ethics Review Committee (Study Code SSHSPH-144).

## Results

A total of thirty-three individuals/institutions were invited to participate in the study. Among them, four declined participation, and no response was received from the remaining twelve. Seventeen respondents were interviewed between October 2021 and April 2022. The composition of the participants included seven participants from pharmaceutical companies, one clinician, one representative from the government HTA agency, four patient group representatives, and four academics (see Table 1). The overall response rate was 51 percent, which may be attributed to participants’ unfamiliarity or disinterest in the topic of discussion and their unavailability. Data saturation was reached after the third interviewee for academics, industry representatives, and patient groups. No new themes emerged within these three clusters, affirming the sufficiency of the sample size.

**Table 1.** Participants characteristics

Characteristics	No. of participants (%) *n* = 17
Role	
Academic	2 (11.76)
Business Unit Lead	1 (5.88)
Clinician	1 (5.88)
Decision maker	1 (5.88)
International expert	2 (11.76)
Market access	6 (35.29)
Patient advocate	4 (23.53)
Institution type	
Academic	4 (23.53)
Government	1 (5.88)
Healthcare provider	1 (5.88)
Industry	7 (41.18)
Patient group	4 (23.53)
Scope of work	
International	5 (29.41)
Regional	2 (11.76)
Local	10 (58.82)
Sex	
Female	10 (58.82)
Male	7 (41.18)

### Summary of Themes

From the interviews, five key themes emerged, reflecting the participants’ overall perceptions and attitudes toward RSAs. A detailed description of each theme is provided in the subsequent sections.

#### Stakeholders perceive RSAs moderately positively despite limited experience

Interpretations and perceptions of RSAs varied among participants, reflecting the diverse nature of these agreements. Despite these differences, most stakeholders acknowledged RSAs as a prospective tool to be employed when circumstances warrant. Notably, those with a robust comprehension of RSAs in line with the ISPOR definition predominantly hailed from academia and industry, especially individuals in roles related to market access or health economics. While patients and clinicians possessed a less comprehensive understanding, their perceptions of RSAs leaned positive when the concept was explained to them.

Among stakeholders, certain individuals regarded RSAs as “a way to improve patient access and reduce uncertainty” (Table 2, quote from Interviewee 6, Industry). Conversely, some expressed skepticism, characterizing RSAs as “not a panacea” (Interviewee 1, Academia) and noting the existence of alternative, less intricate payment models. Despite these reservations, there were compelling rationales behind the interest in outcomes-based risk-sharing agreements (OBRSAs). Stakeholders identified potential benefits such as improved patient access, reduced uncertainty, and the ability to “demonstrate the value of innovation” (Interviewee 8, Industry).

**Table 2.** Key themes and illustrative quotes

Theme	Description	Illustrative quotes
Stakeholders perceive RSAs moderately positive despite limited experience	While the levels of understanding and experience on RSAs varied, they were perceived positively by the majority of stakeholders due to their potential ability to improve access to patients and reduce uncertainty in assessments	“I don’t think that there would be people that would be against it. I just think that it would be… It’s just something that hasn’t been done yet. And I think a lot of what we are looking at currently is not in that space. But for new drugs, I don’t see why not” (Interviewee 2, Academic) “An outcomes–based agreement could be one of the fair features to ensure earlier patient access while even giving the manufacturers an opportunity to demonstrate the value of their innovation” (Interviewee 6, Industry)
There is a need for clarity of objectives and transparent criteria for implementation	The utility of RSAs can vary and may only be appropriate in certain circumstances, as other options are available. The objective for pursuing RSAs need to be made explicit and known to stakeholders, and strong justification when doing so	“In terms of the specific type of innovations and circumstances where this will make sense. That, to me, is the most important question to answer. Because ultimately, right, outcomes–based payment is a means not an end. It’s not the end in itself. And the question is, what does it solve? Can we solve these things with other solutions? And those are important questions that we need to ask ourselves” (Interviewee 6, Industry)
The current data infrastructure is both a barrier and a facilitator	Immature data collection infrastructure is the most commonly cited barrier to implementation. A robust data monitoring system is a necessary condition so as not to put too much administrative burden on clinicians and other key stakeholders	“Singapore needs a conducive environment that enables a collection of high quality, real–world data easily and efficiently, in a timely manner, to enable such innovative approach the payment scheme to be implemented in Singapore” (Interviewee 7, Industry) “Conceptually, I feel these are very data intensive and would require established and rigorous data collection frameworks that allow the collection of patient level data to a high degree of fidelity” (Interviewee 17, Government Agency)
Stakeholder engagement, mutual trust, and co–creation are necessary	Lack of trust between industry and government is cited as one of the key barriers to implementation. The success of RSAs heavily relies on mutual trust between stakeholders and their ability to align goals; hence, more avenues and channels for communication and collaboration are desired	“I think the perception is that it needs to be more of a partnership, rather than a kind of I tell you what to do kind of approach, which is the current perception” (Interviewee 11, Industry) “I think, is that we need a very good relationship between industry and the health technology assessment sector, there really needs to be a platform for people to discuss transparently, and for these arrangements to be made. And for people to have faith in the execution” (Interviewee 2, Academic)
Broader capacity building is a priority	Many stakeholders are still unfamiliar with the concept or intent of such schemes and will require a lot of capacity building. There is a need to prioritize areas where there is a higher chance of success and a lower failure rate	“I would start small and simple and try and learn from a modest scheme. So you work with a group that’s interested in collaborating, you find an obvious problem, you find an easy solution, do good stakeholder engagement, you evaluate along the way, do good research along the way” (Interviewee 1, Academic)

In summary, although RSAs might not be universally applicable, they are perceived as promising tools under specific circumstances. This reflects the recognition that while not suitable for all scenarios, RSAs, specifically outcome-based ones, hold the potential for addressing certain uncertainties and improving health outcomes.

#### There is a need for clarity of objectives and transparent criteria for implementation

Stakeholders underscored that while OBRSAs offer a valuable strategy, they might not always be the most suitable choice. As pointed out by an industry respondent (Interviewee 10), “simpler mechanisms such as upfront price discounts are much easier to implement.” This is also in line with the government agency’s position that less complex schemes such as discounts are preferred, as they pose no burden to clinical stakeholders.

To ensure the efficacy of these agreements, particularly OBRSAs, it is paramount to clearly delineate the objectives of various risk-sharing schemes and establish explicit criteria for their implementation. This level of clarity would provide companies with precise guidance on whether ACE considers these schemes acceptable. Concurrently, aligning incentives among all parties involved in the agreement is of utmost importance, as highlighted by one respondent (Interviewee 7, Industry).

Implementing OBRSAs in Singapore poses unique challenges due to the relatively small population and corresponding market size. This context demands a robust justification for pursuing such schemes, considering the substantial investment needed from pharmaceutical companies. Moreover, stakeholders also observed that the HTA agency appears to lack appetite for such schemes, believing that “existing measures suffice” (Interviewee 5, Healthcare provider) and that there are “no compelling reasons” (Interviewee 10, Industry) to initiate any form of OBRSA.

In this landscape, the initiation and stance of payers play a pivotal role in charting the course for these agreements. Their decisions and signals are critical in defining the trajectory and viability of such arrangements.

#### The current data infrastructure is both a barrier and a facilitator

Introducing novel arrangements or mechanisms such as OBRSAs can lead to notable administrative burdens, especially when data collection is involved. For instance, clinicians may encounter added procedural steps, such as completing separate forms to validate the company’s eligibility for payment based on OBRSAs criteria. This verification requires supporting documentary evidence demonstrating the achievement of the clinical outcome that triggers the subsidy, such as treatment response or progression through diagnostic tests. However, the hurdle of furnishing evidence could be alleviated through the enhancement of information technology (IT) systems capable of promptly and efficiently calculating incentives or rebates (Table 2, quote from Interviewee 7, Industry). This would eliminate the need for supplementary paperwork.

Currently, the “lack of a national integrated system” (Interviewee 3, Academic) stands out as a significant impediment to data access, but ongoing efforts are targeted at refining and streamlining this infrastructure. To ensure an effective system, stakeholders emphasized the need for a rigorous data collection framework (Table 2, quote from Interviewee 17, Government Agency). Furthermore, during the interviews, questions were raised regarding the accountability for data access, funding sources, and the entities that would have access to the data. There is also a shared sentiment that stakeholders “must have faith in the integrity of the data and the system” (Interviewee 2, Academic).

However, efforts to enhance the data collection framework primarily focus on upgrading existing systems designed for routine structured data, which might not be entirely suitable for capturing the clinical outcomes that activate incentives. In summary, although implementing new mechanisms with data collection components can pose administrative challenges, a robust data infrastructure, coupled with appropriately harmonized data collection processes, can effectively mitigate these obstacles.

#### Stakeholder engagement, mutual trust, and cocreation are necessary

Establishing mutual trust stands as a pivotal factor for the effective implementation of RSAs between the government and stakeholders, where “more of a partnership” is desired, rather than “I tell you what to do kind of approach” (Table 2, quote from Interviewee 11, Industry). To achieve this, stakeholders emphasized the need for a “co-owned process by the people” (Interviewee 1, Academic; Interviewee 16, Patient Group) where the government engages in open communication with multiple stakeholders and stressed an emphasis on collective involvement and shared commitment, described as “there has to be some skin in the game for everyone” (Interviewee 2, Academic).

This recurring theme aligns with findings from the systematic review conducted by the authors on OBRSAs. The review underscored the significance of stakeholder engagement in comprehending the needs and objectives of all parties involved, particularly payers and manufacturers. Industry representatives acknowledge increased efforts and channels for engagement with ACE, yet they identify room for further enhancement.

Moreover, the presence of staunch advocates within the medical and patient communities is indispensable for successful execution. This is especially crucial since clinicians will play a pivotal role in implementing OBRSAs, and patients are the primary beneficiaries. Patient groups wish to influence the chosen outcomes and consequently seek substantial participation in the outcomes’ selection and the scheme’s design process.

#### Broader capacity building is a priority

While various stakeholders exhibit varying degrees of familiarity with the local HTA process and the underlying rationale and objectives of RSAs, there is a consensus that more comprehensive capacity-building efforts are needed. Stakeholders recognize the potential benefits of learning from the experiences of HTA bodies in other countries, such as the United Kingdom’s National Institute for Health and Care Excellence (NICE) and Australia’s Pharmaceutical Benefits Advisory Committee (PBAC). Experience sharing is seen as a valuable approach, facilitating the exchange of insights and strategies to address diverse stakeholder concerns (Interviewee 9, Industry). It is notable that ACE draws inspiration from and integrates features of these two systems into local policies.

Proposals have emerged to initiate pilot projects at a smaller scale before embarking on national-level implementations. This phased approach aims to “start simple and learn from a modest scheme,” as indicated by an academic respondent (Table 2, quote from Interviewee 1). The suggestion is to begin within a specific cluster or regional health system to gain practical insights and refine the approach before broader adoption. Additionally, a participant from academia proposed commencing with a disease or therapeutic area that carries a lower risk of failure. This strategic approach minimizes the potential political repercussions in the case of setbacks.

Furthermore, an important emphasis centers on the enhanced involvement of patients and the recognition of their perspectives in shaping mechanisms such as risk sharing. This recognition reflects the growing acknowledgment of the value of incorporating patient insights and preferences into the design and execution of healthcare policies.

### Candidate Disease Area or Therapy

Stakeholders emphasized that risk-sharing schemes hold promise “in circumstances where data are insufficient, causing high uncertainty” (Interviewee 2, Industry). These scenarios, such as those found in oncology or rare diseases, present an ideal backdrop for the application of these schemes. Additionally, stakeholders stressed that these schemes should be reserved for expensive and potentially unaffordable technologies, particularly those where there are challenges in patient access.

The scope of technology suitable for risk-sharing schemes was also discussed. Stakeholders advocated for an inclusive approach, suggesting that such schemes should not solely pertain to drugs or therapeutics. Instead, they proposed considering other high-cost technologies such as diagnostics and medical devices (Interviewee 17, Government Agency). However, it was recommended to initiate these schemes with drugs initially due to the broader familiarity and experience in this domain (Interviewee 6, Industry).

Various risk-sharing arrangements were proposed, with a focus on OBRSAs, including schemes such as money-back guarantees and conditional treatment continuation. These mechanisms are especially relevant in scenarios where there is uncertainty on treatment outcomes, whether success or failure. In terms of financial arrangements, the suggestion of treatment caps was put forth. These caps could be tied to the units of the drug used or the total costs incurred. This approach is typically suitable when uncertainty surrounds the benefits after a certain duration of treatment, such as during a maintenance phase. Nevertheless, it would still require linkage with reliable data on utilization and or additional criteria for treatment continuation.

While the specifics of operational aspects should align with the particular drug or disease area, stakeholders converged on two overarching principles for scheme design. First, the scheme must be “equitable to all stakeholders” (Interviewee 14, Patient Group). Second, it needs to be “flexible enough to accommodate those who fall outside the normal treatment parameters” (Interviewee 5, Healthcare provider). These guiding principles underscore the importance of fairness and adaptability in designing risk-sharing schemes that effectively address uncertainties and diverse patient needs.

## Discussion

Through this qualitative study, we find that most of Singapore’s relevant stakeholders are interested in exploring outcome-based RSAs as an alternative mechanism to pay for select technologies where traditional schemes may not be appropriate. Stakeholders believe this will improve patient access, foster innovation, and address uncertainty in the data. However, certain barriers, such as lack of explicit criteria, data infrastructure, and stakeholder engagement, need to be addressed before implementing such schemes in the local context. This research also highlights the need for broader capacity building of institutions and individuals on HTA and health financing to enhance the capabilities of multiple stakeholders involved, including the industry, clinicians, patient groups, and academia.

The barriers highlighted by stakeholders in this qualitative inquiry align with those reported in published reviews and qualitative studies (21–24). A review of the European experience highlighted that interest in RSAs increased alongside the push for value-based pricing and cost containment but eventually plateaued due to difficulties in implementing and evaluating RSAs, leading to a shift toward simpler financial-based schemes (24). The study also affirms the lack of necessary infrastructure as a barrier and suggests that the capacity of available staff and IT systems must be considered when assessing the appropriateness of RSAs (24;25). Similarly, a qualitative study by Bosch in 2019 involving Dutch stakeholders found that industry representatives were most optimistic about RSAs but lacked sufficient power to change policy directions (26). In contrast, healthcare payers (health insurers in the Dutch system) were less enthusiastic but instrumental in spearheading efforts around outcome-based schemes. A Catalan-specific paper documenting their RSA echoes findings from this study, identifying “appropriate financial, technical, and administrative resources, and strong stakeholder commitment and communication” as facilitators of successful implementation (27). The proposed solutions in these publications align with those provided by the participants in this study, but obtaining insights from stakeholders with a deeper knowledge and understanding of Singapore’s healthcare system adds value.

Our study contributes to a scarce body of literature documenting country-specific experiences and perceptions regarding RSAs. While the findings may not be generalizable to other settings, as is typical with qualitative research, they can be considered valuable by government agencies, industry stakeholders, and other groups affected by these decisions. Furthermore, our study suggests that substantial effort is needed to build capacity in HTA among various stakeholder groups, including industry representatives. This study adds to the growing body of evidence supporting HTA policy development and capacity building in Singapore. We believe that the lead researcher maintained objectivity throughout the interviews and data analysis, providing an external viewpoint of the healthcare system as a foreign academic (PhD student) rather than as a user or provider within the system, with no conflicts of interest.

Despite these strengths, the study is not without limitations. We acknowledge the relatively low response rate from invited participants, which may have limited the breadth of the findings. It would have been beneficial to have more representation from other healthcare payers, such as private insurance companies, government agencies, clinicians, and healthcare providers (e.g., hospital administrators). However, we reached thematic saturation within the stakeholder groups with more than one interviewee, and recurring insights were observed among these clusters. Future capacity-building activities and research should focus on raising awareness among a broader group of stakeholders and uncovering insights not captured in this study.

However, careful examination of tradeoffs is necessary, as planning and executing outcome-based RSAs require substantial effort. If such schemes are pursued in Singapore in the future, it becomes imperative to strengthen the existing data collection infrastructure for effective monitoring. Additionally, enhancing stakeholder engagement, particularly by the government agency, and fostering mutual trust are essential prerequisites for successful agreements. This necessitates more opportunities for dialogue and feedback involving the agency, the industry, clinicians, and patient groups. Moreover, to build trust among stakeholders, there should be increased transparency in the processes. Recognizing potential disparities in perspectives, a commitment to broader capacity building is indispensable for strengthening the overall HTA system and facilitating innovative financing mechanisms in Singapore. To achieve this, stakeholders could benefit from peer-to-peer learning from other countries. Furthermore, targeted sessions aimed at improving technical skills and information campaigns for clinicians and patients should be considered as part of the investment in capacity building.

## Supporting information

Bayani and Wee supplementary material Bayani and Wee supplementary material

## Supplementary material

The supplementary material for this article can be found at http://doi.org/10.1017/S0266462324000217.
