# Supplementary material for: Value-based payment for high-cost treatments in Singapore: a qualitative study of stakeholders’ perspectives
Source: Int J Technol Assess Health Care. 2024 Apr 17;40(1):e22. doi: 10.1017/S0266462324000217 (PMC11569909; doi:10.1017/S0266462324000217)
Supplement: Bayani and Wee supplementary material [file S0266462324000217sup001.docx]

**Interview Guide: Value-based payment for high-cost treatments**

Introduction

Self-Introduction

My name is _. I am from NUS SSHSPH and I will be conducting the interview for the study today. First of all, I want to thank you for taking the time to attend to this interview today.

Purpose of interview

The purpose of this interview is to understand your views on a value-based payment model in Singapore. We are interviewing relevant stakeholders who may have a stake in such a model when explored/implemented.

Audio Recording

I will be taking some notes during the session as we speak. But I would not be able to write everything that is covered during the interview. So, with your permission, we would like to record this interview, are you okay with that <Mr/Mrs [last/name]>?

Verification of informed consent

We would like to confirm that you have read the Participant Information Sheet and provided consent via the e-consent/physical form that we have sent on [specify date signed]. Kindly restate verbally that you have read the PIS and you agree to participate in this study.

Timing of Interview

Thank you for agreeing to proceed with this interview, which will take around 40 to 60 minutes of your time.

Confidentiality & Privacy

All responses and recordings will be kept confidential. This means that your interview responses will only be accessed by the research team members. We will ensure that any information we include in our report does not identify you personally. The transcripts and recordings will be password protected as well.

Because we’re on tape, please be sure to speak up so that we don’t miss any of your comments. The audio recordings will be used in writing up the interview, to ensure the write-up is accurate and complete.

Permission and Participation

Your participation in this interview is completely voluntary. Remember, you are free to withdraw from the interview at any point and not respond to any questions you don’t wish to answer.

Do you have any question so far?

Can I have your permission to begin this interview?

## ━━━━━━━━━━━━━━━━━━━━━━━━━━━▲━━━━━━━━━━━━━━━━━━━━━━━━━━━

We will first ask the main thematic questions and let the participants respond and share. Probing questions will only be asked after the participant has shared, depending on what he/she has shared.

| **Theme** | **Subthemes/Probing questions** |
| --- | --- |
| 1. **Can you please introduce yourself, and describe your role or function?** | 1. How long have you been working in your current role? 2. Have you always worked locally / Do you have experience in working in other health systems? |
| 1. **Today, we are going to discuss value or outcomes-based payment schemes. Have you heard of this term before?** | 1. Are you familiar or have you ever heard of any of the terms “value or outcomes-based payment”, “risk-sharing agreement”, or “managed-entry agreements?” 2. Where have you come across the term before? 3. Do you have any experience of working on such a scheme in the past?   *If respondent says no to #1, explain definition using PBRSA ISPOR TF Report highlighting the 5 key components, risk-sharing between manufacturer and payer.*  *1) there is a program of data collection agreed between the manufacturer and the payer*  *2) data collection is done following regulatory approval*  *3) price, reimbursement or revenue for the product or technology are linked to the outcome of the data collection mechanism*  *4) the data collection is intended to address uncertainty about efficacy or effectiveness of the product and lastly*  *5) these arrangements provide a different distribution of risk between the payer and the manufacturer*  *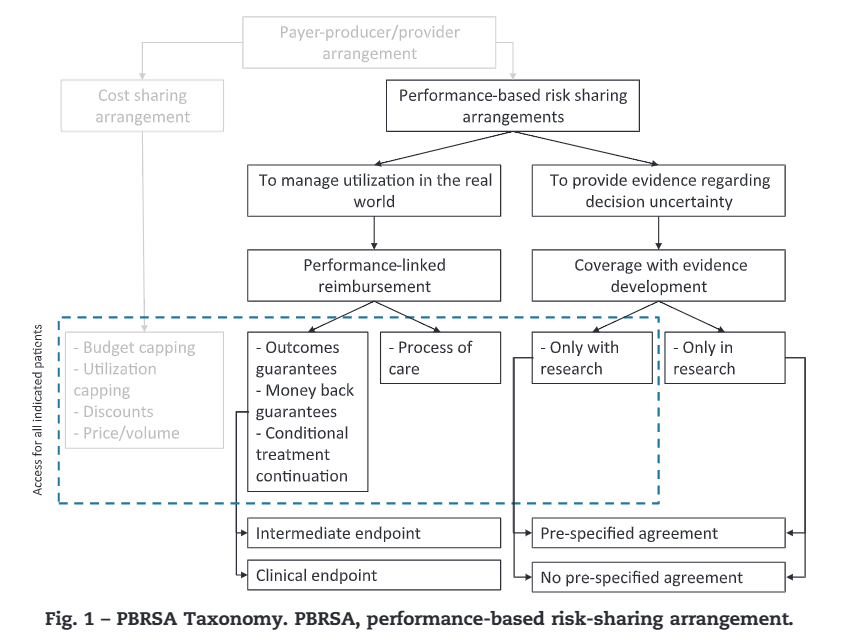*  *Specific examples:*   - *Bortezomib for multiple myeloma in UK NICE: conditional treatment continuation (stopping rule after 4 cycles) and outcomes guarantee (rebate for non-responders), done to mitigate negative consequences of uncertainty about product value* - *Coverage with evidence development for bosentan for PAH, collected data through registry*   *If the respondent said yes, clarify further to know what s/he meant by VBC.* |
| 1. **From your perspective, is there any interest to explore such a scheme in Singapore context?** | 1. What are your general thoughts about the idea of outcomes-based schemes? 2. Do you think there is interest / positive sentiment around it in Singapore context? If yes, proceed with #4 (conducive factors) first. If no, proceed with #3 (barriers) first. 3. If there is interest, can you describe how an ideal scheme would look like? (Probe: candidate treatment, outcomes, payment arrangement or type of agreement) 4. What would make such a scheme acceptable to you? What are the necessary characteristics? (Imagine there are no barriers for now.) |
| 1. **In reality, we recognize that there are constraints and barriers to feasibility. What do you foresee as the key barriers to its implementation in Singapore context?** | 1. What do you think is/are the main reason/s why Singapore has not implemented such schemes? Note that most developed countries have implemented some form of performance-based risk-sharing.   *Probes:*   - *Fragmented multi-payer system makes it too complicated* - *Nothing wrong with the current system of paying for cancer drugs* - *Government doesn’t see it as a priority or is risk averse* - *Limited capacity to collect data, conduct needed analyses to identify sources of uncertainty* - *Transaction costs/evidence collection cost are too high (i.e. the system is not set up to collect data for this purpose)* - *Significant resource / cost burden to stakeholders involved (e.g. data collection is expensive)* - *Significant effort required to execute agreements compared to simple discounts (e.g. paperwork is too troublesome)* - *Difficult to operate under conditions with limited transparency (e.g. commercial confidentiality)* - *Difficult to reach mutual trust and understanding* - *Challenging to identify/define meaningful outcomes and measuring them in real-world settings* - *Limited evidence that it actually improves access and health outcomes* - *Other reasons (expound)* |
| 1. **Do you think Singapore is ready to explore outcomes-based payments? Why or why not?** | 1. What do you think are the necessary conditions or conducive factors for Singapore to adopt risk-sharing agreements?   *Probes:*   - *More patients are pushing for access to potentially beneficial drugs (responding to patient demand)* - *Drug costs are very high and government should act on it* - *There is leadership commitment now to make healthcare more accessible* - *Physicians are more receptive to the idea now* - *PBRSAs improve equity* - *Prior experience from other settings can be used as a starting point* - *We are more equipped now given our data infrastructure* - *Better acceptability of real-world data sources and evidence among stakeholders (clinicians, payers)* - *Other reasons (expound)* |
| 1. **Do you think that outcomes-based payment is applicable to all health technologies and services** | 1. If we were to implement risk-sharing agreements in Singapore, which area should we focus on? It is only innovative drugs? How about diagnostics, medical devices or healthcare services delivery models? |
| 1. **If this scheme were to be explored further in the near future (3-5 years), how do you think stakeholders should prepare and build capacity to sustain it?** | 1. Which stakeholder do you think holds the most burden / who should be responsible in making this scheme possible? Options: government, pharmaceutical company, clinicians, academics, patients, etc. 2. What are the needed requirements to make such models a possibility?   *Probes:*   - *Better acceptability at the system level (identify stakeholder)* - *More knowledge about various schemes and implementation requirements* - *Data infrastructure (e.g. analytics, epidemiology and economic data) for measuring and monitoring relevant outcomes* - *Timely collection of reliable data* - *More staff with strategic, business-oriented thinking* - *Need to fully understand clinical value of treatments* - *Better model for incentives 🡪 how can we subsidize given that payer (Medisave/Medshield), PHI, patient, etc. pays?* |
| 1. **Wrap-up** | 1. Do you have anything else that you would like to share? |

Thank you. We have come to the end of the interview. Your sharing has been most helpful, and we thank you for taking time. We will share a summary report after we have completed all the interviews.
